# A Lizard Predation Experiment Demonstrates Batesian Ant Mimicry in *Camponotus lateralis*

**DOI:** 10.1002/ece3.74163

**Published:** 2026-08-13

**Authors:** Herbert C. Wagner, Felix Kraker, Heinrich Römer, Kristina M. Sefc

**Affiliations:** ^1^ Institute of Biology University of Graz Graz Austria

**Keywords:** aposematism memory, *Crematogaster scutellaris* group, Hymenoptera: Formicidae, intraspecific color morphs, Italian wall lizards (*Podarcis siculus*), myrmecophagy

## Abstract

The Mediterranean ant species *Camponotus lateralis* is often associated with the unpalatable *Crematogaster scutellaris* complex, and workers of both taxa commonly share trails. The recent discovery of three color morphs in *Ca. lateralis*, each resembling sympatric *Crematogaster* model species, provides context for the hypothesis of Batesian mimicry. Here, a feeding experiment was conducted using 25 Italian wall lizards (*Podarcis siculus*) as real predators. Our experiment tested four predictions regarding the predation risk for inaccurate mimics, accurate mimics, and models. Each lizard individual was confronted either with *Cr. scutellaris* or *Cr*. *schmidti* models, which both have a reddish head and a blackish gaster but differ by the former having a blackish and the latter a reddish mesosoma. Minor ant workers of *Ca. lateralis*, mimicking each one of these models, were used as inaccurate and accurate mimics. Also, the jet‐black *Ca. piceus* was used as an inaccurate mimic. Lizards ingested 1.8 times more *Camponotus* than *Crematogaster* ants. Without model experience, lizards ingested 1.9 times more inaccurate and accurate mimics than after gaining such experience. Predation on model ants increased 1.4‐fold when models were spatially closer to the mimics, a result consistent with the expectation that positive experience with mimics weakens avoidance of models through stimulus generalization. Although lizards ingested similar numbers of inaccurate and accurate mimics, 2 months after model experience, they chose the inaccurate mimic first in 83% of cases. We conclude that resemblance and spatial proximity to the model decrease predation risk for the mimic and increase predation risk for the model. We suggest that predation drives the evolution of aposematism in *Crematogaster* and Batesian color mimicry in *Ca. lateralis*. Conversely, myrmecophagy could have contributed to the evolution of cognitive abilities in lizards.

## Graphical abstract

Adaptiveness of ant mimicry was tested in an experimental terrarium with eight glass bowls with three ants each using lizards as real predators (dotted lines show holes; full lines show barriers). Bowls 1, 2, 7, and 8 were stocked with inaccurate and accurate mimics, Bowls 3–6 with the model. This experimental design was used to test four predictions regarding the predation risk for inaccurate mimics, accurate mimics, and models.

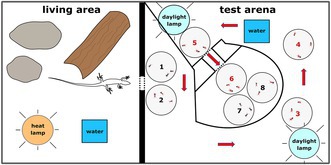

## 1 Introduction

Aposematism is an anti‐predator strategy in which organisms of low profitability to be consumed—like poisonous or well‐defended species—evolve conspicuous visual, chemical, or other signals. The theory predicts that predators associate aversive experiences with these signals and, consequently, learn to avoid aposematic individuals (Poulton 1890, 1909; Cott 1940; Endler 1988; Schmidt 1990; Tseng et al. 2014; Caro and Ruxton 2019). Batesian mimicry is a widespread strategy that takes advantage of aposematism by imitating these signals without evolving the defensive characteristics that make aposematic organisms unprofitable (Bates 1861; Fisher 1930; Wickler 1971; Quicke 2017).

Many ant species defend themselves against predators with inedible chemicals, stingers, or spines (Cavill and Robertson 1965; Maschwitz 1975; Attygalle and Morgan 1984; Schmidt 1990; Laurent et al. 2004; Ito et al. 2016); some of them exhibit aposematic colors (Wilson 1988; Lenoir and Dejean 1994; Merrill and Elgar 2000; Jones et al. 2004; Pekár et al. 2017, 2022). Also, the conspicuous color patterns of unpalatable *Crematogaster* ants have been suggested to be aposematic (Ito et al. 2004; Marlier et al. 2004; Billen et al. 2011; Schifani et al. 2022; Wagner 2025; Wagner and Csősz 2025; Wagner et al. 2025), which allows predators to discriminate them especially from other similarly sized ants (Pérez‐Delgado and Wagner 2024; Kraker and Wagner 2025). There are a little bit more than 20 examples of ants mimicking other ant species (Wagner et al. 2025). In most studies, ant mimicry in ants has been suggested based on investigations of color, morphology, or distribution (Forel 1874, 1891; Santschi 1919; Bernard 1967; Ward 1984; Gobin et al. 1998; Merrill and Elgar 2000; Powell et al. 2014; Rasoamanana et al. 2017; Zettel et al. 2018; Fisher and Peeters 2019; Wagner 2019; Wagner and Csősz 2025). Very often, ant mimicry in ants is associated with interspecific trail‐sharing of the mimic and the model (Ito et al. 2004; Ward 2009; Wild and Brake 2009; Gallego Ropero and Feitosa 2014; Menzel et al. 2014; Fisher and Bolton 2016; Schifani et al. 2022, 2024; Pérez‐Delgado and Wagner 2024; Wagner et al. 2025).

**FIGURE 1:**
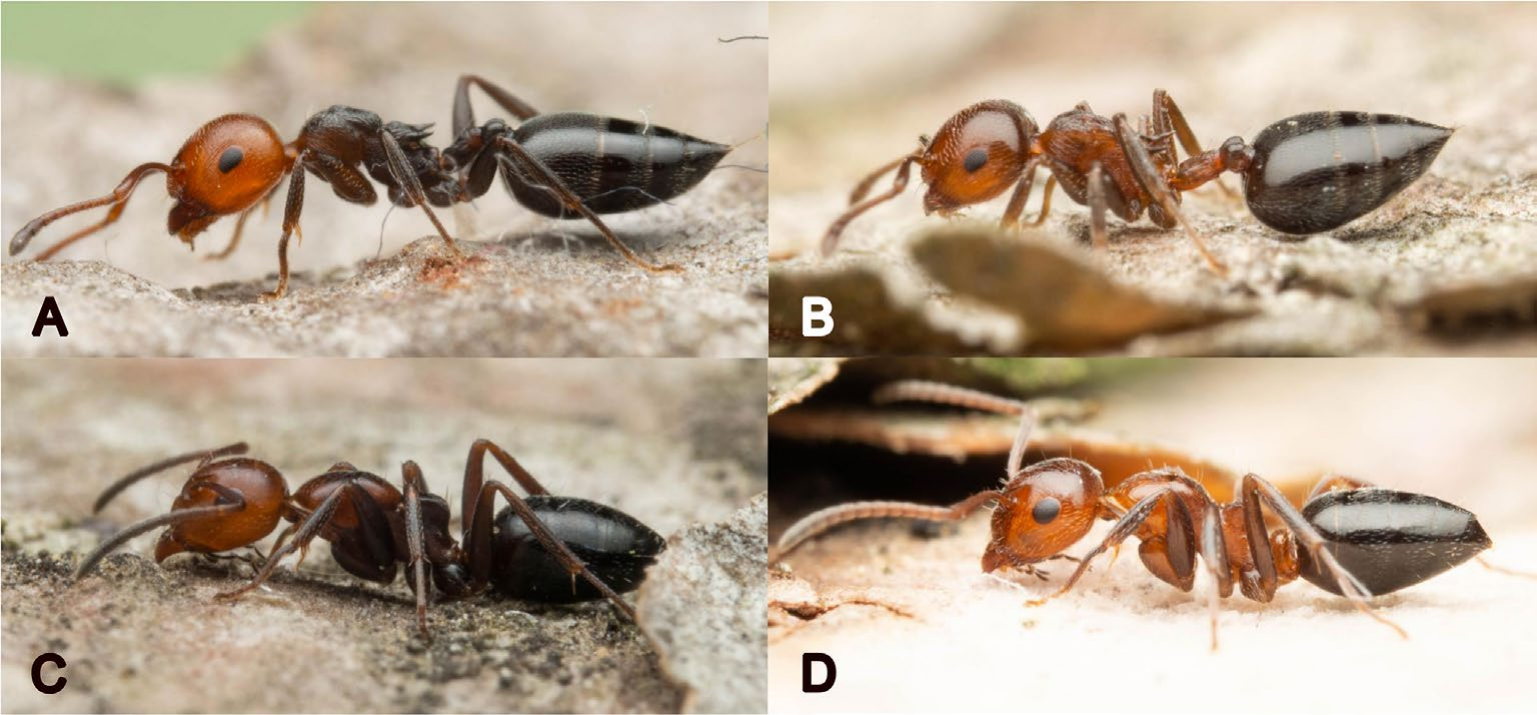
Workers of *Crematogaster scutellaris* (A; model), *Cr. schmidti* (B; model), *Camponotus lateralis* with a blackish mesosoma (*scutellaris*‐like color morph; C; mimic), and *Ca. lateralis* with a reddish mesosoma (*schmidti*‐like color morph; D; mimic).

Workers of the Mediterranean ant *Camponotus lateralis* frequently follow trails of species in the *Crematogaster scutellaris* complex (Emery 1915; Goetsch 1942, 1951, 1953; Kaudewitz 1955; Baroni Urbani 1969; Carpintero et al. 2005; Menzel et al. 2010; Stukalyuk and Radchenko 2011; Schifani et al. 2022; Stukalyuk et al. 2023; Wagner et al. 2025; Csősz et al. 2026). It has been shown that they mimic various sympatric *Crematogaster* species in terms of color. For example, *Ca. lateralis* has often a reddish head, a blackish mesosoma, and a blackish gaster in Italy, where it occurs sympatrically with *Cr. scutellaris*, which has the same color pattern. In contrast, *Ca. lateralis* has a reddish head, a reddish mesosoma, and a blackish gaster in most parts of the Balkans, where it co‐occurs with the similarly colored *Cr. schmidti* (Wagner 2014; Seifert 2018, 2019; Kraker and Wagner 2025). From now on, we refer to these morphs of *Ca. lateralis* as *scutellaris*‐like and *schmidti*‐like color morphs (Figure 1).

Color mimicry in *Ca. lateralis* has been suggested to be Batesian already long before regional color morphs were detected (Emery 1886; Müller 1923; Soulié 1960; Passera and Aron 2005; Menzel et al. 2010). Recently, a study investigating feces of the lizard *Podarcis muralis* showed that, among ant workers, *Cr. scutellaris* and *Ca. lateralis* were strongly underrepresented in relation to their trophic availability. This imbalance was interpreted as a consequence of unpalatability and aposematism in *Cr. scutellaris* and Batesian mimicry and/or trail sharing in *Ca. lateralis* (Wagner 2025). However, the unpalatability of *Cr. scutellaris* for vertebrate predators has not been tested in a large‐scale experimental study (Marlier et al. 2004; Wagner 2025).

While experiments have been applied to test the adaptive value of ant mimicry in spiders and beetles (Palmgren et al. 1937; Taniguchi et al. 2005; Nelson and Jackson 2006; Nelson et al. 2006; Nelson 2012; Ramesh et al. 2016; Shamble et al. 2017; Pekár et al. 2020; Veselý et al. 2021; Hölldobler and Kwapich 2023), we are aware of only one example in which ant mimicry of ants was tested experimentally with predators: Ito et al. (2004) used chicks of domestic chicken as predators to demonstrate that *Camponotus* sp. is a Batesian mimic of *Crematogaster inflata* , both with a yellow‐black color pattern. Naïve chicks ingested *Camponotus* sp. but spat out *Cr. inflata*. Subsequently, they also no longer attacked *Camponotus* sp., although they still attacked the ant species *Anoplolepis gracilipes* , which has a distinctly different color and shape.

In a preliminary observation, a single Italian wall lizard (*Podarcis siculus*) preferred the jet‐black *Camponotus piceus* , an edible species widespread in the Mediterranean, to the closely related but mimetic *Ca. lateralis* after experience with *Cr. scutellaris* (Wagner 2013, 2014, 2025). This observation prompted the hypothesis that predation by lizards drives the evolution of mimicry in *Ca. lateralis* (Wagner et al. 2025). To test this hypothesis, we conducted a feeding experiment with *P. siculus*. This lizard species has previously been shown to discriminate between aposematic and non‐aposematic carabid beetles (Bonacci et al. 2008). In addition, anecdotes suggest that it ingests ants but avoids *Crematogaster* also in the field (Wagner et al. 2025). Moreover, fecal‐pellet and stomach‐flushing analyses yielded remains of *Camponotus* and *Crematogaster* (Bombi and Bologna 2002). Its distribution area (Kwet 2022) is fully embedded within those of *Ca. lateralis* and its *Crematogaster* model‐species (Lebas et al. 2016; Seifert 2018) and it often occurs syntopically with these ants (Wagner et al. 2025). Taken together, these observations identify *P. siculus* as an appropriate test predator. From the hypothesis that Batesian mimicry in *Ca. lateralis* is adaptive to reduce lizard predation, we derived the following predictions:

- Prediction 1: Lizards ingest more ants of the mimic ( *Camponotus lateralis* group) than of the model ( *Crematogaster scutellaris* complex).
- Prediction 2: Experience with the model (*Crematogaster*) reduces consumption of the mimic (*Camponotus*).
- Prediction 3: Experience with the mimic (*Camponotus*) increases consumption of the model (*Crematogaster*). This prediction examines whether spatial association with mimics influences predator responses to the model. Such a pattern would be consistent with our hypothesis that Batesian mimicry weakens the aposematic signal and thus predator avoidance of the model (Wickler 1971; Kikuchi and Pfennig 2013).
- Prediction 4: After experience with the model (*Crematogaster*), lizards prefer inaccurate over accurate mimics (*Camponotus*). Lizards experienced with *Cr. scutellaris* are expected to prefer the *schmidti*‐like color morph of *Ca. lateralis* over the *scutellaris*‐like one, and vice versa, lizards experienced with *Cr. schmidti* should prefer the *scutellaris*‐like color morph over the *schmidti*‐like one.

## 2 Material and Methods

### 2.1 Animals

Ants of the four species *Ca. lateralis* (95 nests), *Ca. piceus* (22), *Crematogaster scutellaris* (48), and *Cr. schmidti* (42) were collected alive (localities in File S1: Sampling localities), kept in plastic boxes with nest material (wood and soil), and fed with honey and mealworms. The ant colonies survived usually for several months, some up to more than 1 year. Ant workers of 102 nests were used in the experiment. Workers of *Cr. scutellaris* (which have a blackish mesosoma) and *Cr. schmidti* (which have a reddish mesosoma) represented two slightly differently colored models. Minor workers of *Ca. lateralis* and *Ca. piceus* represented the mimics. The colors of *Ca. lateralis* individuals were classified as *scutellaris*‐like and *schmidti*‐like based on human visual perception; published red‐green‐blue data from workers of the same nests (Kraker and Wagner 2025) were available for comparison. We defined *Ca. lateralis* ants sharing the mesosoma color (i.e., either reddish or blackish, Figure 1) with the model as accurate mimics, and those *Ca. lateralis* ants differing in the mesosoma color from the model as well as those of the jet‐black species *Ca. piceus* as inaccurate mimics.

The Italian wall lizard (*P. siculus*) was used as a predator. Twenty naïve lizards were bought from Megazoo Alpha GmbH (Leipzig; born in spring 2022; File S2: Captive‐bred certificate). Five further lizards used in the study were their offspring born in spring 2023, resulting in a total of 13 males and 12 females. Lizards had an age of 0.5–2.5 years during the experiment; five lizards died during the interval of the experiment. Lizards were kept individually or pairwise in terraria and fed mainly with domestic crickets and mealworms, sometimes with *Chorthippus* grasshoppers, adult flies, apples, earthworms, common daisy, tipulids, isopods, spiders, earwigs, and pears. Domestic crickets and mealworms were dusted with ready‐to‐use vitamin and mineral powders before being put into the terraria (Reptivite Reptile Vitamins, Zoo Med Laboratories, USA; TRIXIE Vitamin‐/ Mineralstoffkomplex, Germany). The experiment was carried out with the ethical approval of the ethics committee of the University of Graz (permit number GZ. 39/88/63 ex 2022/23) (File S3: Ethics approval).

### 2.2 Experimental Terrarium

**FIGURE 2:**
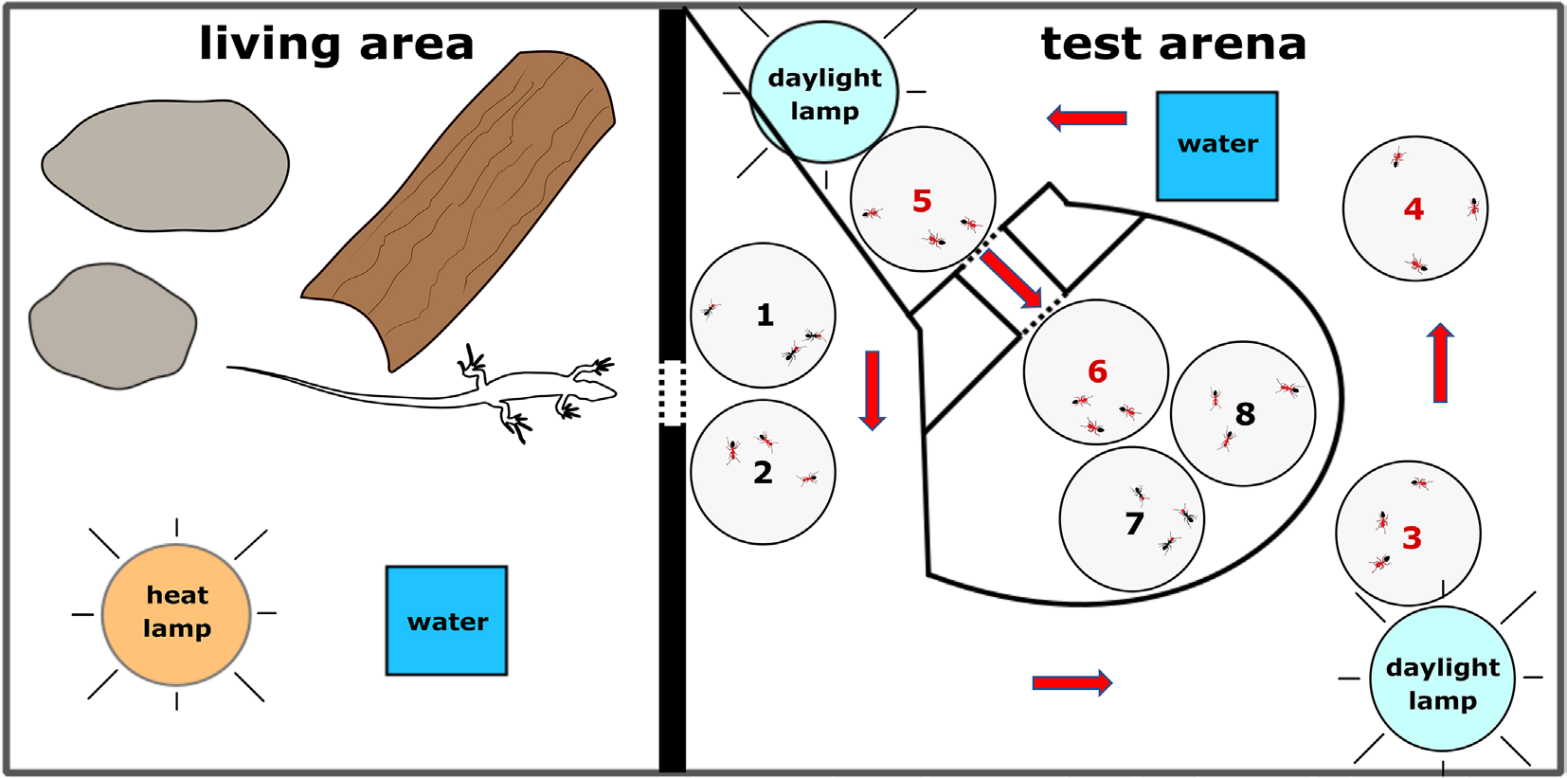
Experimental terrarium with living area and test arena, connected by a hole (dotted lines). Full lines show barriers. The test arena contained glass Bowls 1–8. The arena center with Bowls 6–8 was accessible via a hole (dotted lines). Each bowl contained three ants. An example case of Run 1 is shown: In Bowls 1 and 7, the *scutellaris*‐like morph of *Camponotus lateralis* (here: The inaccurate mimic); in Bowls 2 and 8, the *schmidti*‐like morph of *Ca. lateralis* (i.e., here: The accurate mimic); and in Bowls 3–6 (red numbers) the *Crematogaster* model (here: *Cr. schmidti*). A lizard walking through the arena first met ants of Bowls 1 and 2, then 3, 4, 5, and finally 6–8.

The experimental terrarium had a size of 60 × 30 × 30 cm and was separated into two areas: the living area (25 × 30 × 30 cm) and the test arena (34 × 30 × 30 cm); both areas were connected by a 3 cm wide hole, the lower margin of which was 4 cm above the arena ground (Figure 2). The living area was equipped with stones and dead wood as hiding places, a heat lamp (2520K, 580 Lumen, CRI = 78, 75 W, E27, Exo‐Terra, China), and a water supply. The arena was illuminated by two full‐spectrum daylight lamps (5400K, 1000 Lumen, 10 W, 220–240 V AC 50/60 Hz, PF ≥ 0.5, CRI = 95, E27; natur‐nah.de, David Communication e.K., Reppenstedt, Germany); the color temperature of 5400 Kelvin simulated natural sunlight conditions as in fore‐ or afternoon (Nassau 1998). A 7‐cm‐high plastic barrier guided the lizards to the consecutive feeder stations in the test arena. Water was offered between feeder stations 4 and 5. The arena was covered by a glass plate placed on top of the plastic barrier. Feeder stations consisted of a total of eight round glass bowls (diameter of 6.0 cm and a depth of 1.8 cm) containing the ants. The arena center with Bowls 6–8 was accessible via a 3 cm wide hole with its lower margin 4 cm above the ground. The bowls were lowered into the arena floor in order to allow the lizards free access into the bowls unhindered by glass walls. All four sides of the terrarium were covered with cardboard on the outside to prevent viewing the surroundings.

### 2.3 Experimental Design

The experiment was conducted from July 2023 to October 2024 and consisted of four runs (Runs 1–4). Before participating in a trial, lizards were not fed for 10 days. Already 20 days prior to a trial, domestic crickets, which are large and energy‐rich, were removed from the diet to prevent the buildup of long‐lasting energy reserves; between 20 and 10 days before a trial, lizards got only eight mealworms per week. The lizards were put individually into the living area of the experimental terrarium 2–3 days before a trial started; they had the opportunity to enter the arena as well. The preparations for trials were performed between 7:30 and 8:30 usually on Mondays, Wednesdays, and Fridays: Between 7:30 and 8:00, when the lizards still were in the living area, an untransparent barrier between the living area and test arena was installed to block access to the arena. Then, glass bowls were washed, the upper half of the bowl walls were covered with ant‐outbreak defense oil (ANTSTORE, Berlin, Germany) to prevent the escape of ants, and each bowl was stocked with three ants of the same nest. The species or color morph of the ants placed in each bowl varied depending on the run and lizard individual (details under Run 1). Between 8:20 and 8:30, the barrier blocking the connection to the arena was removed to allow the lizard access to the arena, and the video recording was activated. Videos were recorded on a smartphone placed above the terrarium. All trials took place from 8:30 to 12:30 (4 h). After each trial, the respective lizard was returned to its home terrarium. Dead and living ants, if present, were removed.

For each trial, we scored the sequence in which the lizards visited the bowls for ant ingestion and the numbers of ingested ants from each bowl from the videos (File S4: Raw data). Ants were defined as ingested only if they were captured and swallowed, rather than spat out. Analyzing only these robust ingestion data made it unnecessary to interpret which modalities lizards putatively used to assess the edibility of ants. In addition to visual observation, 24 of 25 lizards used tasting with the tongue in exceptional cases as an alternative strategy to identify ants.

The following paragraphs describe Runs 1–4, all of which adhered to the general procedure outlined above. Runs 1–3 addressed all four predictions, whereas Run 4 addressed only Prediction 4 (overview in Table 1).

**TABLE 1:** Information on Runs 1–4 showing the number of lizards, the ants placed in the bowls (mimic or model), and the evaluated predictions. One of mimic Bowls 1 and 2 contained the inaccurate and the other the accurate mimic; the same applied to mimic Bowls 7 and 8. The inaccurate mimic was in Runs 1 and 2 a color morph of *Ca. lateralis* differing from the color of the model, whereas in Runs 3 and 4 it was the black *Ca. piceus*. The model was *Cr. scutellaris* for half of the lizards and *Cr. schmidti* for the other half throughout the experiment.

	Number of lizards	Bowls 1 and 2 (mimic bowls)	Bowls 3–6 (model bowls)	Bowls 7 and 8 (mimic bowls)	Evaluated
Run 1	25	Mimic (accurate/inaccurate)	Model	Mimic (accurate/inaccurate)	Predictions 1–4
Run 2	24	Mimic (accurate/inaccurate)	Mimic	Mimic (accurate/inaccurate)	Predictions 1–4
Run 3	21	Mimic (accurate/inaccurate)	Model	Mimic (accurate/inaccurate)	Predictions 1–4
Run 4	20	Mimic (accurate/inaccurate)			Prediction 4

Run 1: Twenty‐five lizards, 13 males and 12 females, were used. Bowls 3–6 (see Figure 2 for the position of bowls in the experimental terrarium) were stocked with the model ants and thus termed “model bowls”; twelve trials (6 male and 6 female lizards) used solely *Cr. scutellaris* and 13 trials (7 male and 6 female lizards) solely *Cr. schmidti* as model. Bowls 1, 2, 7, and 8 contained the mimics and were thus termed “mimic bowls.” Mimic bowls were arranged as two pairs, Bowls 1 and 2 plus Bowls 7 and 8, each comprising one bowl with the *scutellaris*‐like and the other with the *schmidti*‐like color morph of *Ca. lateralis* (Figure 2). Mimic placement in the mimic bowls was randomized among four possible combinations (*scutellaris*‐like mimic in Bowls 1 and 7, 1 and 8, 2 and 7, or 2 and 8), ensuring that each combination was used equally often in each lizard sex. In lizards with *Cr. scutellaris* as model, the *scutellaris*‐like ants of *Ca. lateralis* were the accurate and the *schmidti*‐like ones the inaccurate mimics; in lizards with *Cr. schmidti* as model the situation was vice versa. The rationale behind this design was to give the lizards the opportunity to choose between different‐colored mimics in Bowls 1 and 2 at the beginning of the trial, when no preference was expected, and then in Bowls 7 and 8, when a preference for the inaccurate mimic was predicted following experience with a model species (*Cr. scutellaris* or *Cr. schmidti*) in the model bowls (Figure 2). Because 13 lizards did not ingest any ants from the mimic Bowls 7 or 8, these trials were repeated once with the same individuals in order to increase sample size for evaluating Predictions 3 and 4, which required data from these bowls. To evaluate Predictions 1 and 2, only data from the first trial per lizard were used.

Run 2, which partly served as a control, was conducted 68 ± 10 days (min = 58, max = 100) after Run 1 with 12 male and 12 female lizards. While in Run 1 half of the males and half of the females had been trained with either *Cr. scutellaris* or *Cr. schmidti* models, in Run 2 all eight bowls were stocked with mimics. The color morphs placed in Bowls 1, 2, 7, and 8 were the same as in Run 1 for each lizard. The aims of this run were to test (i) for position effects of bowls and (ii) whether lizards still responded to their experience with any *Crematogaster* color after > 60 days.

Run 3 was conducted 79 ± 28 days (min = 31, max = 132) after Run 2 with 10 male and 11 female lizards. The set up was similar to Run 1, but the inaccurate mimic of Run 1 was replaced by *Ca. piceus*, a jet‐black and thus even more clearly inaccurate “mimic”, to facilitate recognition by the lizards. The model was the same as in Run 1 for each lizard. The positions of accurate and inaccurate mimics in bowls 1, 2, 7, and 8 were identical to those in Run 1 for each lizard. Because 12 lizards did not ingest any ants from the mimic bowls 7 or 8, these trials were repeated once with the same individuals in order to increase sample size for evaluating Predictions 3 and 4, which required data from these bowls. To evaluate Predictions 1 and 2, only data from the first Run 3 trial per lizard were used.

Run 4 was conducted 68 ± 11 days (min = 60, max = 91) after Run 3 with 10 male and 10 female lizards. Bowls 1 and 2 were stocked with mimics of the same color morph and species as in Run 3 for each lizard, while the six remaining bowls remained empty. The aim of this run was to test whether lizards avoided accurate mimics after their experience with the model > 60 days back (Prediction 4).

### 2.4 Statistical Analysis

Data were analyzed with generalized linear mixed‐effects models (GLMMs) in R (R Core Team 2024) using the packages lme4 (Bates et al. 2015), dplyr (Wickham et al. 2014), MASS (Ripley et al. 2013), and emmeans (Lenth 2016).

To compare numbers of ingested ants, we fitted GLMMs with binomial error distributions, including “lizard ID” as a random effect variable and total number of ants per bowl (3 ants) as model offset. Predictor variables were “bowl” (nominal variable), “run” (nominal variable), or “mimic” (nominal variable with levels “accurate” and “inaccurate”), as required by the tested prediction. Models were fitted separately for the individual predictions. Regression coefficients estimated by the GLMM are reported as *β*(…), that is, the change in the log‐odds of being ingested, which represents the effect of switching from the reference level of the binomial predictor to the non‐reference level (specified in the parentheses), along with *z* values and *p* values testing the statistical significance of the change. Additionally, we converted the estimated log‐odds into probabilities of being ingested for each predictor level and plotted them along with their 95% confidence intervals.

Prediction 1 was tested by modeling the number of ingested ants from mimic versus model bowls as response variable and “bowl” as predictor, separately for Runs 1, 2, and 3 (File S5: R scripts). Prediction 2 was tested by modeling the number of ingested mimic ants from Bowls 1 + 2 or Bowls 7 + 8 as response variable and “run” as predictor, comparing Runs 1 versus 2 and Runs 3 versus 2 separately. Prediction 3 was tested by modeling the number of ingested ants from Bowls 5 and 6 as response variable and “bowl” as predictor, separately for Runs 1, 2, and 3. Finally, Prediction 4 was tested by modeling the number of ingested inaccurate versus accurate mimics from Bowls 1 + 2 or 7 + 8 as response variable and “mimic” as predictor. In one case (evaluating whether inaccurate mimics were ingested more often than accurate mimics in Bowls 7 + 8 in Run 3), the binomial model did not converge; therefore, we used a Poisson error distribution to model the number of ingested ants per bowl (Pekár and Brabec 2016).

We used binomial tests to compare the number of times a lizard first fed from one bowl to the number of times it first fed from the other bowl to the expectation of equal proportions (*p* = 0.5) to evaluate whether the first attack targeted inaccurate mimics more often than accurate mimics (Prediction 4; Bowls 7 and 8 in Runs 1 and 3; Bowls 1 and 2 in Runs 2 and 4; File S5: R scripts). A few lizards did not avoid the model and were, consequently, not expected to respond to the associated aposematic or mimetic coloration. Hence, analyses excluded trials with lizards which ingested at least 75% of all model ants (≥ 9 model ants) in the respective run (Runs 1 and 3) or—if no model was used in this run (Runs 2 and 4)—the run before (Runs 1 and 3). By doing so, six and five lizards in Runs 1 (and 2) and 3 (and 4), respectively, were excluded. Two‐sided tests were used in all analyses.

### 2.5 Exclusion of A Priori Preferences for Ant Color and Position

To support the interpretation of our results, we tested whether lizards showed a priori preferences for ant color or position in the arena. Using binomial tests, we compared the number of times a lizard first fed from one bowl versus the other to the expectation of equal proportions (*p* = 0.5). This allowed us to assess whether lizards selected randomly between differently colored ants when starting their trial (Bowls 1 and 2 in Runs 1 and 3) and whether they showed preferences for certain bowls (Bowls 1 vs. 2 or 7 vs. 8 in Runs 1–3 and 1 vs. 2 in R4; Bonati et al. 2008). Feeding from Bowls 1 and 2 in Run 1, 12 lizards first ingested a *scutellaris*‐like mimic, while the other 12 lizards first ingested a *schmidti*‐like mimic (*p* = 1). Similarly, in Run 3, 8 lizards first ingested *Ca. piceus* and 10 lizards first ingested *Ca. lateralis* (*p* = 0.8145). We also did not detect a preference to feed from a particular bowl: In Runs 1–4, lizards first targeted Bowl 1 in 42 trials and Bowl 2 in 40 trials (*p* = 0.9122); similarly, in Runs 1–3, lizards first targeted Bowl 7 in 16 trials and Bowl 8 in 19 trials (*p* = 0.7359).

## 3 Results

### 3.1 Prediction 1: Lizards Ingested More Ants of the Mimic ( *Camponotus lateralis* Group) Than of the Model ( *Crematogaster scutellaris* Complex)

**FIGURE 3:**
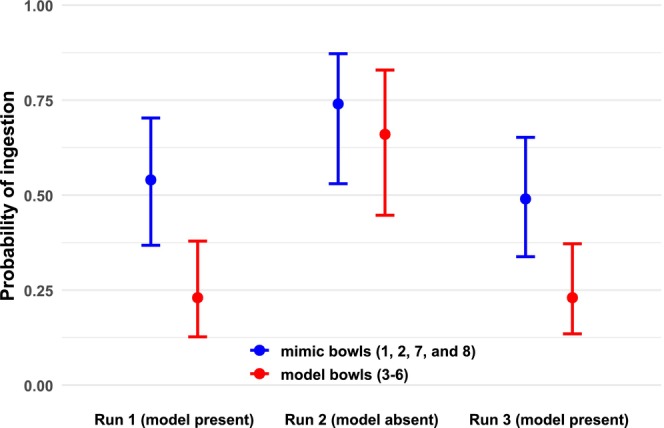
Ants of the mimic ( *Camponotus lateralis* group) were more likely to be ingested than ants of the model ( *Crematogaster scutellaris* complex). Modeled estimates (File S5: R scripts) of ants ingested from the mimic bowls (1, 2, 7, and 8) and the model bowls (3–6) are shown. Note: In Run 2, the control run, both mimic and model bowls were stocked with mimic ants. The dots represent the estimated probabilities; the whiskers represent the 95% confidence intervals.

In Runs 1 and 3, lizards ingested more *Camponotus* than *Crematogaster* ants; the differences were highly significant (Run 1: *β*(Bowls 3, 4, 5, and 6) = −1.37, *z* = −6.483, *p* = 8.99e^−11^, *n* = 25; Run 3: *β*(Bowls 3, 4, 5, and 6) = −1.17, *z* = −5.425, *p* = 5.79e^−8^, *n* = 21; Figure 3). These differences were not due to preferences for certain positions of bowls in the experimental setup: In Run 2, where all bowls were stocked with *Ca. lateralis*, lizards did not ingest significantly different numbers of ants from mimic and model bowls (*β*(Bowls 3, 4, 5, and 6) = −0.34, *z* = −1.567, *p* = 0.1170, *n* = 24, Figure 3; empirical data in File S6: Empirical results).

### 3.2 Prediction 2: Experience With the Model (*Crematogaster*) Reduced Consumption of the Mimic (*Camponotus*)

**FIGURE 4:**
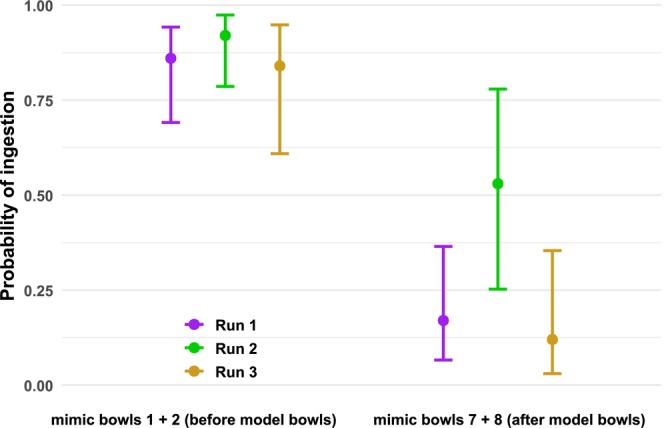
Consumption of the mimic (*Camponotus*) was less likely after experience with the model (*Crematogaster*) than without this experience. Modeled estimates of mimics ingested from Bowls 1 + 2 and 7 + 8 by lizards in Run 1 to 3 are shown. In Run 2, the control run, mimic ants ingested from Bowls 7 + 8 after mimic (not model) consumption from model bowls is shown. The dots represent the estimated probabilities, the whiskers the 95% confidence intervals.

Numbers of mimic ants ingested from Bowls 1 + 2 in Runs 1 and 3 were slightly lower than those in Run 2 (*β*(Run 1) = −0.56, *z* = −1.527, *p* = 0.126642, *n* = 25; *β*(Run 3) = −0.90, *z* = −2.256, *p* = 0.0241, *n* = 21; Figure 4). In contrast, after lizards had experienced the model in Runs 1 and 3 (offered in model Bowls 3–6), they ingested highly significantly fewer mimics from bowls 7 + 8 than in Run 2, where all bowls were stocked with mimics (*β*(Run 1) = −1.67, *z* = −4.899, *p* = 9.64e^−7^, *n* = 25; *β*(Run 3) = −2.14, *z* = −5.285, *p* = 1.26e^−7^, *n* = 21; Figure 4; File S6: Empirical results).

### 3.3 Prediction 3: Experience With the Mimic (*Camponotus*) Increases Consumption of the Model (*Crematogaster*)

**FIGURE 5:**
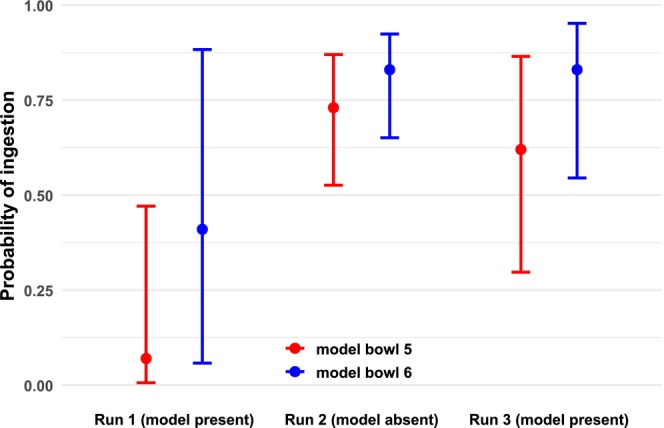
Model ants were more likely to be ingested in spatial proximity to the mimics. Modeled estimates of ants ingested from model Bowls 5 (farther away from the mimic) and 6 (closer to the mimic) by lizards in Runs 1 to 3 are shown. In Runs 1 and 3, model bowls (3–6) were stocked with model ants; in Run 2, the control run, model bowls were stocked with mimic ants. The dots represent the estimated probabilities; the whiskers the 95% confidence intervals.

Only those lizards that fed from mimic Bowl 7 and/or 8 were considered; that is, 17 lizards in Run 1 and 13 lizards in Run 3. The numbers of model ants ingested from Bowl 5, which was distant from those stocked with mimics, were compared with those ingested from Bowl 6, which was close to mimics in Bowls 7 and 8. Lizards ingested more ants from Bowl 6 than from Bowl 5 (Run 1: *β*(Bowl 6) = 2.23, *z* = 4.272, *p* = 1.946e^−5^, *n* = 17; Run 3: *β*(Bowl 6) = 1.09, *z* = 2.518, *p* = 0.0118, *n* = 13; Figure 5). In the control run, Run 2, where no *Crematogaster* ants were offered, lizards did not ingest significantly more *Camponotus* ants from Bowl 6 than from Bowl 5 (*β*(Bowl 6) = 0.56, *z* = 1.510, *p* = 0.131, *n* = 15; Figure 5; File S6: Empirical results).

### 3.4 P4: After Experience With the Model (*Crematogaster*), Lizards Preferred Inaccurate Over Accurate Mimics (*Camponotus*)

**FIGURE 6:**
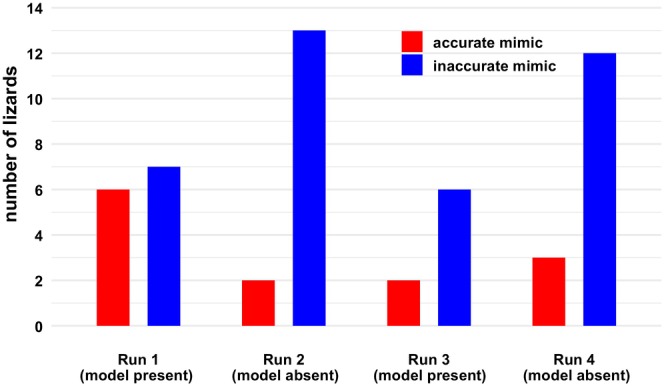
Lizards with model (*Crematogaster*) experience preferred inaccurate over accurate mimics. First mimic‐ingestion events of lizards, under exclusion of those individuals which had ingested at least 75% of all model ants (≥ 9 workers), in Runs 1–4 are shown. In Runs 1 and 3, the situation in Bowls 7 and 8 is shown (directly after model experience); in Runs 2 and 4, the situation in Bowls 1 and 2 is shown (> 2 months after model experience). In Runs 1 and 2, the inaccurate mimic was represented by *Ca. lateralis*, in Runs 3 and 4 by *Ca. piceus*.

In Runs 1 and 3, lizards ingested nearly identical numbers of ants of the inaccurate and the accurate mimic that were offered in Bowls 7 and 8, indicating that lizards had not learned to discriminate against accurate mimics (Run 1: *β*(inaccurate) = −1.308e^−6^, *z* = 0.000, *p* = 1.0, *n* = 19; Run 3: *β*(inaccurate) = −2.687e^−6^, *z* = 0.000, *p* = 1.0, *n* = 16). A similar result was obtained when the same lizards were tested > 60 days later (Run 2: *β*(inaccurate) = 0.6798, *z* = 0.989, *p* = 0.3226, *n* = 18; Run 4: *β*(inaccurate) = 4.499e^−6^, *z* = 0.000, *p* = 0.999994, *n* = 15; File S6: Empirical results). Likewise, there was no preference to target inaccurate mimics first when lizards had experienced the model in the same run (Bowls 7 + 8; Run 1: 7 lizards first ingested the inaccurate and 6 the accurate mimic; *p* = 1.0; Run 3: 6 lizards first ingested the inaccurate and 2 the accurate mimic; *p* = 0.2890625). However, when tested > 60 days after encountering the model, most lizards targeted the inaccurate mimic first (Bowls 1 + 2; Run 2: 13 lizards first ingested the inaccurate and 2 the accurate mimic, *p* = 0.007385254; Run 4: 12 lizards first ingested the inaccurate and 3 the accurate mimic, *p* = 0.03515625; Figure 6; data without exclusion of those individuals which had ingested at least 75% of all model ants (≥ 9 workers) in File S6: Empirical results).

## 4 Discussion

The main findings of our laboratory feeding experiment with *P. siculus* are the following:

- Prediction 1: Lizards ingested significantly more ants of the mimic ( *Camponotus lateralis* group) than of the model ( *Crematogaster scutellaris* complex). This demonstration of lower profitability in model consumption is a basic requirement for the mimicry hypothesis.
- Prediction 2: Lizards ingested significantly more inaccurate and accurate mimics when naïve to the models than after gaining experience with them.
- Prediction 3: Lizards ingested significantly more model ants when they were spatially closer to the mimics.
- Prediction 4: More than 2 months after experience with models, most lizards turned to the inaccurate mimic first but ended up ingesting similar numbers of inaccurate and accurate mimics.

### 4.1 Prediction 1: Lizards Ingested More Ants of the Mimic ( *Camponotus lateralis* Group) Than of the Model ( *Crematogaster scutellaris* Complex)

Although the numbers of ingested *Camponotus* ants were significantly greater than those of *Crematogaster* ants, the differences appear to be smaller than one would expect between palatable and unpalatable ants. The large number of ingested unpalatable ants might be a result of the hunger the lizards had after 10 days without food. Moreover, the simultaneous offering of both genera in close physical proximity in our design could have led to identification errors due to mimicry and spatial proximity and thus to a convergency of ingestion cases between the two genera: While some *Crematogaster* ants can be assumed to be ingested due to a confusion with *Camponotus*, some *Camponotus* ants can be assumed to be avoided due to a confusion with *Crematogaster* (see also Section 4.3 | Prediction 3 for this argumentation). Under natural conditions, nevertheless, where *Ca. lateralis* workers represent 2.4% of *Crematogaster* workers in shared trails (Wagner et al. 2025; in Runs 1 and 3 *Camponotus* individuals comprised 100% of the number of *Crematogaster* individuals), consumption of *Crematogaster* (and also of *Ca. lateralis*) is lower, as the chance for a lizard is higher to gain negative experience with *Crematogaster* than positive experience with *Camponotus*. A recent study investigating feces of *P. muralis* from a *Cr. scutellaris* territory found that lizards ingested, in comparison with trophic availability, 22 and 2558 times more workers of *Ca. lateralis* and *Ca. piceus*, respectively, than of *Cr. scutellaris* (Wagner 2025). The described difference between mimic and model consumption in our experiment versus the situation in the field is well in line with the role of relative abundances: It is a general pattern that mimetic species are rarer than their models (Huheey 1984; McIver and Stonedahl 1993), leading to the assumption that mimicry can only evolve in systems in which models are distinctly more frequent than their mimics (Wickler 1971; Lindström et al. 1997; Kikuchi and Pfennig 2010). A Batesian mimic, generally, is under negative frequency‐dependent selection (Kunte et al. 2021). In *Ca. lateralis*, we consider small colony size (Stukalyuk and Radchenko 2011; Lebas et al. 2016; Borovsky et al. 2022; Wagner et al. 2025)—in relation to the much larger colonies of the *Crematogaster* model species (Atanassov and Dlussky 1992; Zakharov 2015; Radchenko 2016; Seifert 2018)—as preadaptation to mimicry (Wagner et al. 2025).

### 4.2 Prediction 2: Experience With the Model Reduced Consumption of the Mimic

Our result that the number of ingested mimics (*Camponotus*) after model experience was smaller than without such experience is comparable with those of a southeast Asian study also investigating a mimicry system including a mimetic *Camponotus* and an aposematic *Crematogaster* species (Ito et al. 2004): *Camponotus* sp. was ingested by chicks of domestic chicken before but not after the experience with the unpalatable *Cr. inflata*. Concerning our results, it is important to mention that not only both color morphs of the mimic *Ca. lateralis* but also the jet‐black *Ca. piceus* were ingested less frequently after the offering of the model than without (see also Section 3.4 | Prediction 4 the non‐significant differences between ingested ant individuals of the accurate mimic of *Ca. lateralis* and *Ca. piceus*). This suggests that spatial proximity to unpalatable *Crematogaster* ants is also advantageous for palatable ants of similar size but different coloration. Similarly, a decreased ingestion of palatable ants of *Dorymyrmex bureni* in the presence of toxic fire ants ( *Solenopsis invicta* ) was shown in experiments with the eastern fence lizard (Phrynosomatidae: *Sceloporus undulatus* )—juvenile individuals of this reptile could not distinguish between two similar but non‐mimetic ant species (Venable and Langkilde 2019; Venable et al. 2019). Following *Crematogaster* trails is also known from blackish species of the *Ca. lateralis* group, like *Ca. piceus* and *Ca. spissinodis* (Forel 1898; Schifani et al. 2022, 2024; Wagner et al. 2025). Field data on ingestion rates of non‐mimetic ants in spatial proximity to *Crematogaster* are lacking. Nevertheless, our laboratory result showing reduced consumption of the non‐mimetic *Ca. piceus* after experience with the model makes us believe that being close to *Crematogaster*, like following its trails, is a preadaptation to mimicry evolution in *Camponotus* (Wagner et al. 2025). The perception of *Crematogaster* trail pheromones (Scarano et al. 2024) in *Camponotus* has already been postulated to be a preadaptation for various parabioses between these two genera (Menzel et al. 2014). The individuality in terms of the number of ants ingested and the memory of the lizards in our study was remarkable: While a few lizards never ingested a single *Crematogaster* ant (but many of *Camponotus*), a few others ingested all 12 available in one trial. Moreover, while a few experienced lizards avoided accurate mimics of *Ca. lateralis* for more than 10 min (but ingested the inaccurate ones immediately or after critical examination), a few others either ingested or avoided all ants without examination. Cognitive variability in reptiles has frequently been observed (Benes 1969; Endler and Mappes 2004; Szabo et al. 2021). It has been argued, interestingly, that variability among predator individuals could more likely promote mimicry evolution: If some predators already avoid strongly imperfect mimics, while others select critically among more accurate ones, each slight step in mimicry evolution should be adaptive by addressing different predator individuals (Wickler 1971; Chai 1986).

### 4.3 Prediction 3: Experience With the Mimic Increased Consumption of the Model

Our results showed that model ants were ingested more often when they were spatially close to the mimics. We repeatedly observed that lizards, which had previously already learned to avoid the model, resumed model consumption after ingesting mimics from adjacent bowls, giving the impression that positive taste experience with the mimic stimulated the lizards to hastily consume further ants (e.g., 1–2 model ants) without critical examination. The observed effect is consistent with predator learning dynamics. Positive experiences with the mimic weaken avoidance responses and, through stimulus generalization, increase the likelihood of attacks on models occurring near mimics (Mostler 1935). Spatial proximity would further reinforce this process by increasing local encounter rates with palatable mimics and thereby the frequency of positive experiences. We conclude that the presence of the mimic increased predation risk for the model. Batesian mimicry is therefore costly for model species, as it dilutes the effectiveness of their aposematic signaling (Wickler 1971; Kikuchi and Pfennig 2013). The diversification of aposematic color patterns in *Crematogaster* may have evolved to prevent confusion with camponotine trail‐followers of ancestral coloration (Kraker and Wagner 2025; Wagner and Csősz 2025).

### 4.4 Prediction P4: After Experience With the Model, Lizards Preferred Inaccurate Over Accurate Mimics

The color differences between inaccurate and accurate mimics did not have an influence on the numbers of ants ingested. However, > 60 days after encountering the model, the lizards ingested inaccurate mimics significantly more often as their first choice than accurate mimics, regardless of whether the inaccurate mimic was another color morph of *Ca. lateralis* or the jet‐black *Ca. piceus*. Consistent with the mimicry concept, lizards experienced with either model species avoided the mimic sharing the same color pattern (i.e., the accurate mimic). In other words: The color of the presented model species determined which mimetic color pattern was the adaptive one.

It is delightful to revisit the old Darwinian question whether natural selection is sufficient to produce adaptive traits (Darwin 1859), in our case to modulate a color pattern into the direction of the model (Bates 1861). A scenario, as constructed in our study—that is, simultaneously offering of inaccurate and accurate mimics at the same distance to the lizard—might not typically be encountered in nature. But we should not forget how frequent these ants are (Gené 1842; Bračko, Gomboc, et al. 2014; Bračko, Wagner, et al. 2014; Vesnić et al. 2017; Kiran and Karaman 2020; Schifani et al. 2022; Bračko 2023; Borowiec et al. 2024): They forage on most sunlit trees in Mediterranean near‐natural biotopes, and most *Crematogaster* trails are followed by *Camponotus* (Wagner et al. 2025). While lizards often watch for prey near *Crematogaster* trails, *Camponotus* workers can quickly escape or fall off the tree after touching a lizard (Wagner et al. 2025). Sometimes, several *Ca. lateralis* colonies with differently colored workers are associated with a single *Crematogaster* colony (Kaudewitz 1955; Kraker and Wagner 2025; Wagner 2025), increasing the chance that workers of different phenotypes use the same trail simultaneously and, consequently, providing an opportunity for natural selection to act: While a lizard would first attack an inaccurate mimic of *Ca. lateralis*, an accurate mimic in spatial proximity could perceive the lizard and escape. Densities of different lacertid‐lizard species were estimated to be ca. 0.24 ± 0.54 lizards/100 m^2^ at 19 localities populated by *Ca. lateralis* from North Italy to Karpathos (Wagner et al. 2025). While our experiment involved only 25 lizards, millions of wild lizards across the Mediterranean likely face mimetic ants under natural conditions every day during the warm season and select them based on several characters, of which color is one. Counting our results and arguments together, it seems likely that lizard predation suffices to drive color evolution in *Ca. lateralis*.

While predation by birds may have initiated the evolution of ant mimicry in spiders (Veselý et al. 2021), we are not aware of any observations of birds eating *Ca. lateralis* (Wagner et al. 2025). However, most bird species at least occasionally feed on ants (Bull et al. 1992; Seifert 2009; Dean and Milton 2018) and have red color vision (Yerkes and Eisenberg 1915; Lashley 1916; Hess 1917; Wood 1925), so they might also play a role. In contrast, we consider invertebrate predators less likely to drive color mimicry in *Ca. lateralis*, because we are not aware of any observations (Wagner et al. 2025) and most invertebrate predators cannot perceive red (Briscoe and Chittka 2001).

### 4.5 Sensory Modalities in *Podarcis siculus*

Lizards were able to use at least taste and visual sensory modalities to discriminate the mimic from the model. Naïve lizards often captured *Crematogaster* and spat it out in most cases immediately; later, they partly learned to discriminate mimic and model ants visually. We sometimes observed that when experienced lizards hesitated between attacking or avoiding an ant, they tasted it with their tongue. After tasting a mimic, they immediately attacked the ant, whereas in cases of the model, they moved on without any further interaction. *Podarcis siculus* exhibits this tasting behavior also in nature (Wagner et al. 2025), which undoubtedly reduces the advantage of optical mimicry. The perception of chemical cues using the tongue is well known from *Podarcis* (Rensch and Eisentraut 1927; Damme and Quick 2001; Cooper et al. 2002; Bonacci et al. 2008). *Crematogaster* workers, when disturbed or attacked, immediately secrete substances of the Dufour's gland and the poison gland, which make their secretion a highly efficient electrophilic contact poison (Buren 1959; Maschwitz and Kloft 1971; Daloze et al. 1987, 1991; Pasteels et al. 1989). It seems likely that the main or only reason for the avoidance of *Crematogaster* was this secretion. Nevertheless, also ant acid of *Ca. lateralis* is not fully ineffective as a defense weapon against lizards: We observed several lizards killing *Ca. lateralis* ants via biting and waiting next to them static for 1–2 min before ingesting them. We never observed a similar biting and waiting behavior with any other food. We interpret this behavior as a strategy to get rid of the ant acid; this small molecule, with a boiling point similar to that of water (Afzelius 1777), might evaporate soon after biting off the poison gland. These observations suggest that *P. siculus* is adapted to prey on formicine ants.

It is remarkable that lizards did remember the aposematic color patterns for more than 2 months. Agamid lizards and skinks can remember aposematic colors or tissue of prey up to about 60 days (Price‐Rees et al. 2011; Ko et al. 2020). For true lizards (Lacertidae), although literature concerning spatial memory is available (Day et al. 1999; Font 2019), information about memory of aposematic color signals—without repeated presentation throughout the experiment—appears to be scarce (Szabo et al. 2021). It should be underlined that both color morphs of the mimic *Ca. lateralis*, which were presented in our experiment, included the same colors of blackish and reddish and just varied in their respective patterns. Consequently, the visual difference was subtle, requiring high discrimination ability from the lizards. The long‐term memory for > 60 days might be the evolutionary consequence of strong selection pressure. *Podarcis* lizards in nature avoid *Crematogaster* but frequently prey on *Camponotus* (Bombi and Bologna 2002; Wagner 2025; Wagner et al. 2025). *Camponotus lateralis* and *Crematogaster* model‐species are already active at temperatures of around 15°C throughout the year (Soulié 1955; Borovsky et al. 2022), while *P. siculus* needs higher temperatures to hunt (Herter 1940; Avery 1978; Van Damme et al. 1990). Consequently, these ants are available throughout the whole active season of *P. siculus* . Since unpalatable *Crematogaster* species and palatable *Camponotus* species belong to the most common ants in the Mediterranean Basin (Zimmermann 1931, 1935; Carpintero et al. 2005; Santini et al. 2007; Lebas et al. 2016; Arcos and García 2024; Borowiec and Salata 2025), the importance of discriminating between these two genera might have promoted high visual and cognitive abilities.

## Author Contributions

**Herbert C. Wagner:** conceptualization (lead), data curation (lead), formal analysis (equal), funding acquisition (lead), investigation (lead), methodology (lead), project administration (lead), resources (lead), software (equal), validation (lead), visualization (lead), writing – original draft (lead), writing – review and editing (lead). **Felix Kraker:** conceptualization (supporting), data curation (supporting), methodology (supporting), visualization (supporting), writing – review and editing (supporting). **Heinrich Römer:** conceptualization (supporting), funding acquisition (supporting), methodology (supporting), writing – review and editing (supporting). **Kristina M. Sefc:** conceptualization (supporting), formal analysis (equal), funding acquisition (supporting), methodology (supporting), resources (supporting), software (equal), writing – review and editing (lead).

## Funding

This research was funded in large part by the Austrian Science Fund (FWF) [10.55776/P35816]. For the purpose of open access, the author has applied a CC BY public copyright license to any Author Accepted Manuscript version arising from this submission.

## Conflicts of Interest

The authors declare no conflicts of interest.

## Supporting information


**File S1:** Sampling localities.

**File S2:** Captive‐bred certificate of 20 lizards from Megazoo Alpha GmbH.

**File S3:** Ethics approval of the ethics committee of the University of Graz (permit number GZ. 39/88/63 ex 2022/23).

**File S4:** Raw data of number and sequence of the lizards' ant consumption from the bowls.

**File S5:** R scripts.

**File S6:** Empirical results (means, SDs, and graphics).

## Data Availability

The data that support the findings of this study (raw data) are provided as Supplementary Files.
