# Supplementary material for: A Lizard Predation Experiment Demonstrates Batesian Ant Mimicry in Camponotus lateralis
Source: Ecol Evol. 2026 Aug 13;16(8):e74163. doi: 10.1002/ece3.74163 (PMC13470880; doi:10.1002/ece3.74163)
Supplement: Supplementary file 1 [file ECE3-16-e74163-s001.pdf]

GZ. 39/88/63 ex 2022/23

Mag. Herbert Christian WAGNER, Bakk. PhD
Institut für Biologie
per E-Mail an Wagner, Herbert Christian (herbert.wagner@uni-graz.at)

Graz, 05.05.2023

BESTÄTIGUNG

Das Forschungsvorhaben **"Untersuchungen zum Raubdruck gegen fressbare und nicht fressbaren Ameisen unterschiedlicher Färbung durch Eidechsen"** des Autors Mag. Herbert Christian WAGNER, Bakk. PhD im Bereich Biologie (Antrag vom 27.03.2023) wurde von der Ethikkommission der Universität Graz auf seine ethische Vertretbarkeit geprüft und für unbedenklich erachtet.

Für die Ethikkommission:

Dr. Manfred Scaria e.h.
(Vorsitzender)

Für die Richtigkeit der Ausfertigung:

Mag. Sigrid Eder
Rechts- und Organisationsabteilung

Mag. Sigrid Eder
Universitätsplatz 3, 8010 Graz, Austria
Telefon: +43 (0) 316 / 380-1052
E-Mail: ethikkommission@uni-graz.at
http://www.uni-graz.at/de/forschen/organisation/ethikkommission/
