# Supplementary material for: A Lizard Predation Experiment Demonstrates Batesian Ant Mimicry in Camponotus lateralis
Source: Ecol Evol. 2026 Aug 13;16(8):e74163. doi: 10.1002/ece3.74163 (PMC13470880; doi:10.1002/ece3.74163)
Supplement: Supplementary file 2 [file ECE3-16-e74163-s006.pdf]

Übergabeformular für nachweispflichtige Tiere

Exemplar für den Kunden

Datum: 22.02.2023

**Verkäufer / alter Besitzer**	**Käufer / neuer Besitzer**
**Megazoo Alpha GmbH**	Name: Mag. Dr. Herbert Christian Wagner
**Am Rondell 6**	Strasse, Nr.: Dietersdorf 7
**12529 Schönefeld**	PLZ, Ort: 8142 Wundschuh / Austria
	Telefon: +43 680 143 4691

deutscher Name	wissenschaftlicher Name	Geburtsdatum	Geschlecht	Kennzeichen / Artenschutznummer
20x Ruineneidechse	Podarcis siculus	2022	0,0,20	

Die vorbezeichneten Exemplare stammen aus unserem legalen Besitz.
Sie sind im gewerblichen Aufnahme- und Auslieferungsbuch eingetragen unter lfd. Nr. 6221 - 6240

Sie sind ☒ Artengeschützt ☐ nicht Artengeschützt

☒ Sie stammen aus rechtmäßiger deutscher Nachzucht bzw. Nachzucht innerhalb der EG
Die Herkunft/Züchter wird auf behördliche Anfrage benannt.

☐ Sie stammen aus rechtmäßiger Einfuhr
Einfuhrgenehmigungsnummer mit Datum ____________
Einfuhrland (nur bei EU-Mitgliedstatt) ____________

Bei nachweispflichtigen Tieren: Beschreibung, Melde- Buch- oder Citesnummer der Elterntiere
Männlich: 1/12, 1571 - 1588
Weiblich: 13/50, 1589 - 1618

Folgende Belege wurden ☐ **im Original** ☒ **in Kopie übergeben**

- ☐ EU-Bescheinigung nach BNatSchG (Cites)
- ☒ Erklärung über Herkunft besonders geschützter Arten (BArtSchV)
- ☐ Zuchtbestätigung des Züchters
- ☐ Einfuhrgenehmigung

Artengeschützte Tiere, die der Meldepflicht unterliegen, müssen umgehend bei der zuständigen Naturschutzbehörde gemeldet werden.
Bei allen anderen artengeschützten Tieren sind die Nachweise dauerhaft aufzubewahren.

übergeben: 22.02.2022 [Unterschrift] angenommen: ____________

Datum, Name Unterschrift Datum Unterschrift
