# Supplementary material for: A Lizard Predation Experiment Demonstrates Batesian Ant Mimicry in Camponotus lateralis
Source: Ecol Evol. 2026 Aug 13;16(8):e74163. doi: 10.1002/ece3.74163 (PMC13470880; doi:10.1002/ece3.74163)
Supplement: Supplementary file 3 [file ECE3-16-e74163-s002.docx]

**A Lizard Predation Experiment Demonstrates Batesian Ant Mimicry in *Camponotus lateralis***

**Herbert C. Wagner | Felix Kraker | Heinrich Römer | Kristina Sefc**

**File S1:** Sampling localities.

| sites | locality | coordinates | biotope | mesosoma color | date |
| --- | --- | --- | --- | --- | --- |
| 1 | Italy: 1.4 km WNE Passirano | 45.6032° N, 10.0477° E, 252 m | mixed-deciduous-forest margin (*Quercus*, *Fraxinus ornus*, *Castanea sativa*, *Robinia pseudoacacia*, *Sambucus nigra*, *Rubus*, *Hedera helix*) | blackish | 24.-25.IV.2023 |
| 2 | Italy: 1.2 km W Custoza | 45.3702° N, 10.7798° E, 109 m | deciduous-forest remnant with *Quercus*, *Fraxinus ornus*, *Populus*, *Robinia pseudoacacia*, *Ailanthus* *altissima*, *Prunus*, *Ficus*, *Ulmus*, *Hedera helix*, *Smilax*. Steep east-slope. | blackish | 22.-23.IV.2023 |
| 3 | Italy: 1.1 km NNW Battaglia Terme | 45.2985° N, 11.7732° E, 14 m | *Morus* alley with *Hedera helix* and *Smilax* | blackish | 21.-22.IV.2023 |
| 4a | Slovenia: 1.6 km NW Ankaran | 45.5908° N, 13.7234° E, 18 m | *Quercus*-*Ulmus* forest with He*HeHHedera helix* | reddish | 17.-19.IV. and 15.X.2023 |
| 4b | Slovenia: 2.8 km NW Ankaran | 45.5913° N, 13.7042° E, 17 m | *Pinus* forest with He*HeHHedera helix* | reddish | 14.-15.X.2023 |
| 5a | Croatia: 3.8 km ESE Novigrad | 45.3066° N, 13.6103° E, 69 m | meadow with *Quercus* trees with *Hedera helix* | blackish | 14.IV.2023 |
| 5b | Croatia: 3.3 km ESE Novigrad | 45.3113° N, 13.6057° E, 16 m | *Pinus* forest in north exposition | blackish | 15.IV.2023 |
| 6 | Croatia: Krk, 1.7 km N Linardići | 45.0889° N, 14.4667° E, 119 m | *Quercus*-*Ulmus* forest with *Hedera helix* | blackish | 12.-13.IV.2023, 12-13.X.2023 |
| 7 | Croatia: 5.5 km NW Pirovac | 43.8476° N, 15.6123° E, 12 m | *Pinus* forest with *Hedera helix* and *Smilax* | blackish | 9.-10.IV.2023 |
| 8 | Croatia: 2.2 km NNW Makarska | 43.3151° N, 17.0020° E, 69 m | *Pinus* forest | blackish | 6.-7.IV.2023 |
| 9 | Croatia: 9.5 km W Orebić | 42.9851° N, 17.0676° E, 27 m | Macchia with *Smilax* | reddish | 4.-5.IV.2023 |
| 10 | Montenegro: 3.8 km SE Petrovac | 42.1836° N, 18.9759° E, 5 m | *Quercus* trees with *Hedera helix* along the street or single trees in gardens or fallow land | reddish | 2.-3.IV.2023 |
| 11 | Albania: 8.0 km W Levan | 40.6748° N, 19.3940° E, 1 m | *Pinus* forest with *Pistacia* and *Quercus* bushes | reddish | 26.-28.V.2023 |
| 12 | Greece: 2.7 km SSE Ladochori | 39.4685° N, 20.2476° E, 9 m | deciduous forest (*Quercus coccifera*, *Olea europaea*, *Pistacia*, *Quercus ithaburensis*, *Genista*, *Smilax*). South-west exposition | reddish | 15.-16.IV.2024 |
| 13 | Greece: 2.0 km SW Araxos | 38.1755° N, 21.3683° E, 2 m | *Quercus*-*Pistacia* forest with *Smilax* | reddish | 12.-13.IV.2024 |
