# Supplementary material for: A Lizard Predation Experiment Demonstrates Batesian Ant Mimicry in Camponotus lateralis
Source: Ecol Evol. 2026 Aug 13;16(8):e74163. doi: 10.1002/ece3.74163 (PMC13470880; doi:10.1002/ece3.74163)
Supplement: Supplementary file 4 [file ECE3-16-e74163-s003.docx]

**Supplement S6**

**A Lizard Predation Experiment Demonstrates Batesian Ant Mimicry in *Camponotus lateralis***

**Herbert C. Wagner | Felix Kraker | Heinrich Römer | Kristina Sefc**

**Empirical results 3.1-3.4**

In **Runs 1** and **3**, lizards ingested more *Camponotus* (mean ± standard deviation across trials: **Run 1**: 6.3 ± 3.5; **Run 3**: 6.0 ± 3.0) than *Crematogaster* (**Run 1**: 3.6 ± 3.7; **Run 3**: 3.4 ± 3.3) ants. In **Run 2**, where all bowls were stocked with *Ca. lateralis*, lizards did not ingest significantly different numbers of ants from mimic (8.1 ± 3.9) and model (7.5 ± 3.8) bowls.

**3.1 | Prediction 1: Lizards Ingested More Ants of the Mimic (*Camponotus lateralis* group) Than of the Model (*Crematogaster scutellaris* group).**

**Figure 3b. Lizards ingested more ants of the mimic (*Camponotus lateralis* group) than of the model (*Crematogaster scutellaris* group).** Numbers of ants ingested from the mimic bowls (1, 2, 7, and 8; always *Camponotus*) and model bowls (3-6; in **Runs 1** and **3** *Crematogaster*, in **Run 2** *Camponotus*). Attention: The red bar in the control run, **Run 2**, shows mimic (not model) ants ingested from model bowls. The thick lines represent the medians, the boxes include data between the quartiles 1 and 3, the whiskers include data between minimum and maximum values by excluding the outliers, the circles represent mild outliers, and the stars extreme outliers.

**3.2 |** **Prediction 2: Experience With the Model (*Crematogaster*) Reduced Consumption of the Mimic (*Camponotus*).**

Numbers of mimic ants ingested from bowls 1 and 2 in **Runs 1** (4.5 ± 1.9) and **3** (4.3 ± 2.4) were similar to those in **Run 2**. In contrast, after lizards had experienced the model in **Runs 1** and **3** (offered in model bowls 3-6), they ingested highly significantly fewer mimics from bowls 7 and 8 than in **Run 2**, where all bowls were stocked with mimics (**Run 1**: 1.8 ± 2.3 ants from bowls 7 and 8; **Run 3**: 1.6 ± 2.3; **Run 2**: 3.3 ± 2.7).

**Figure 4b. Experience with the model (*Crematogaster*) reduced consumption of the mimic (*Camponotus*).** Numbers of mimics ingested from bowls 1 + 2 and 7 + 8 by lizards in **Run 1** to **3**. In **Run 2**, the control run, all bowls were stocked with mimics. Attention: Mimic bowls 7+8 of the control run, **Run 2** (green bar), shows mimic ants ingested after mimic (not model) consumption from model bowls.

**3.3 | Prediction 3: Lizards Ingested More Ants of the Model (*Crematogaster*) When These Were Spatially Closer to the Mimics (*Camponotus*).**

The numbers of model ants ingested from bowl 5, which was distant from those stocked with mimics (**Run 1**: 0.8 ± 1.3, **Run 3**: 1.6 ± 1.4), were compared with those ingested from bowl 6, which was close to mimics in bowls 7 and 8 (**Run 1**: 1.5 ± 1.4, **Run 3**: 2.1 ± 1.0). Lizards ingested more ants from bowl 6 than from bowl 5. In the control run, **Run 2**, where no *Crematogaster* ants were offered, lizards did not ingest significantly more *Camponotus* ants from bowl 6 (2.3 ± 0.8) than from bowl 5 (2.0 ± 1.2).

**Figure 5b. Lizards ingested more ants of the model (*Crematogaster*) when these were spatially closer to the mimics (*Camponotus*).** Numbers of ants ingested from model bowls 5 and 6 by lizards in **Runs 1** to **3**. In **Runs 1** and **3**, bowls 3-6 were stocked with ants of the model; in **Run 2**, the control run, model bowls were stocked with ants of the mimic.

**3.4 |** **P4: After Experience With the Model (*Crematogaster*), Lizards Preferred Inaccurate Over Accurate Mimics (*Camponotus*).**

In **Runs 1** and **3**, lizards ingested nearly identical numbers of ants of the inaccurate (**Run 1**: 1.1 ± 1.2, **Run 3**: 1.1 ± 1.4) and the accurate mimic (**Run 1**: 1.1 ± 1.2, **Run 3**: 1.1 ± 1.3) that were offered in bowls 7 and 8. A similar result was obtained when the same lizards were tested > 60 days later (**Run 2**: 2.4 ± 1.0 inaccurate and 2.2 ± 1.3 accurate mimics; **Run 4**: 2.5 ± 0.7 inaccurate and 2.5 ± 0.8 accurate mimics).

Likewise, there was no preference to target inaccurate mimics first when lizards had experienced the model in the same run (bowls 7 + 8; **Run 1**: 8 lizards first ingested the inaccurate and 8 the accurate mimic; *p* = 1.0; **Run 3**: 7 lizards first ingested the inaccurate and 2 the accurate mimic; *p* = 0.1796875). However, when tested > 60 days after encountering the model, most lizards targeted the inaccurate mimic first (bowls 1 + 2; **Run 2**: 14 lizards first ingested the inaccurate and 6 the accurate mimic, *p* = 0.1153183; **Run 4**: 15 lizards first ingested the inaccurate and 5 the accurate mimic, *p* = 0.04138947).

**Figure 6b**. **Lizards with model (*Crematogaster*) experience preferred inaccurate over accurate mimics.** First mimic-ingestion events of lizards, without exclusion of those individuals which had ingested at least 75% of all model ants (≥ 9 workers), in **Runs 1**-**4** are shown. In **Runs 1** and **3**, the situation in bowls 7 and 8 is shown (directly after model experience), in **Runs 2** and **4**, the situation in bowls 1 and 2 is shown (> two months after model experience). In **Runs 1** and **2**, the inaccurate mimic was represented by *Ca. lateralis*, in **Runs 3** and **4** by *Ca. piceus*.
